# Supplementary material for: Development of a Protocol for Anaerobic Preparation and Banking of Fecal Microbiota Transplantation Material: Evaluation of Bacterial Richness in the Cultivated Fraction
Source: Microorganisms. 2023 Dec 1;11(12):2901. doi: 10.3390/microorganisms11122901 (PMC10745795; doi:10.3390/microorganisms11122901)

Supplementary material 1: manuscript "Protocol for anaerobic preparation and banking of fecal microbiota transplantation material: evaluation of bacterial richness in the cultivated fraction" by B. Bosch, A. Hartikainen, A. Ronkainen, F. Scheperjans, P. Arkkila, R. Satokari in the special issue "Impact of Fecal Microbiota Transplantation on Human Health 2.0" of section "Gut Microbes" in the Journal Microorganisms

Stool collection donor protocol

1. Collect the sample by following the faecal donor instructions.
2. Write down on the clean container sticker your initials, date, and hour of sampling. Use the waterproof pen provided.
3. Place the container in the sealing plastic bag.
4. Switch on the sealing machine and open the lid by pressing the two side buttons at once.
5. Open the anaerobe indicator strip package and deep one strip in a cup of tap water. Do not touch the reaction test stick zone (blue colour).
6. Place the strip in the sealing plastic bag.
7. Remove the anaerobic gas generation sachet from the cover package and place it last in the sealing plastic bag. Do not open the anaerobic gas generation package until you are ready to seal the bag.
8. Release one side of the container's lid.
9. Introduce the upper part of the sealing bag (around 5 cm) inside the sealing machine.
10. Low down the sealing machine lid until you hear two 'clicks' from both sides, press and release the 'Only Seal' red button and wait until the light turns off. That indicates that the bag will be sealed.
11. Rise the lid up by pressing the two side buttons at once. A sealing line must be shown in the plastic bag. Remove the bag carefully from the machine and switch it off.
12. After sealing, wait 15 minutes and then close the container's lid by pressing gently from the outside.
13. Hand in the sample as soon as possible (preferably 2 hours) to the laboratory facilities.

**Do not store the sample in the fridge or freezer*

**Always use the sealing bags and sealing machine provided*

**After adding the anaerobic gas generator sachets and sealing, some heat will be released from the plastic bag.*

**The video demonstration is attached to the protocol for clearer instructions*

Sealing machine

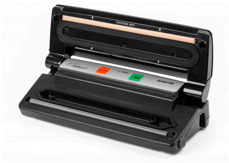

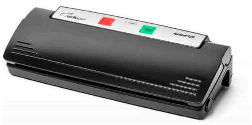

Clean container

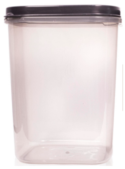

Sealing plastic bag

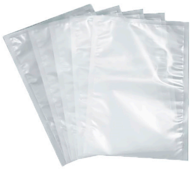

Anaerobe indicator strips

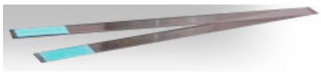

Oxygen scavenger

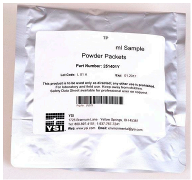

Supplement: Supplementary file 1 [file microorganisms-11-02901-s001.zip › microorganisms-2684343-Supplementary material 1.pdf]
