# Supplementary material for: Development of a Protocol for Anaerobic Preparation and Banking of Fecal Microbiota Transplantation Material: Evaluation of Bacterial Richness in the Cultivated Fraction
Source: Microorganisms. 2023 Dec 1;11(12):2901. doi: 10.3390/microorganisms11122901 (PMC10745795; doi:10.3390/microorganisms11122901)
Supplement: Supplementary file 1 [file microorganisms-11-02901-s001.zip › microorganisms-2684343-Supplementary material 3.pdf]

Supplementary material 3: manuscript "Protocol for anaerobic preparation and banking of fecal microbiota transplantation material: evaluation of bacterial richness in the cultivated fraction" by B. Bosch, A. Hartikainen, A. Ronkainen, F. Scheperjans, P. Arkkila, R. Satokari in the special issue "Impact of Fecal Microbiota Transplantation on Human Health 2.0" of section "Gut Microbes" in the Journal Microorganisms

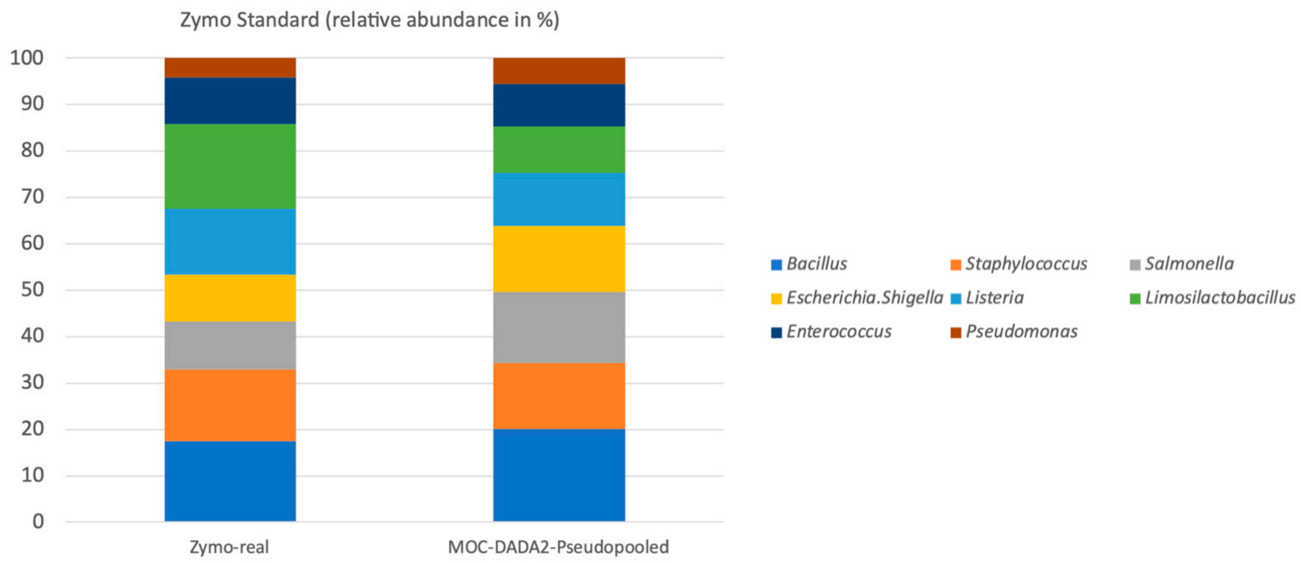

Figure S1. Microbial community DNA Zymo standard. Relative abundance is expressed as percentage (%).
